# The role of radiotherapy for pancreatic malignancies: a population-based analysis of the SEER database

**DOI:** 10.1007/s12094-021-02671-0

**Published:** 2021-07-04

**Authors:** Y. Luo

**Affiliations:** Department of Radiotherapy Oncology, Hainan Cancer Hospital, Affiliated Cancer Hospital of Hainan Medical College, No. 6, Changbin West 4th Street, Xiuying District, Haikou, 570100 Hainan China

**Keywords:** Pancreatic cancer, Prognosis, Surgery, Radiotherapy

## Abstract

**Background:**

To investigate the role of adjuvant radiotherapy in patients with pancreatic cancer.

**Methods and patients:**

The patients with pancreatic cancer from 18 registered institutions in the Surveillance Epidemiology and End Results (SEER) database were retrospectively analyzed. The characteristics of patients who would benefit from adjuvant radiotherapy were screened, as well as whether neoadjuvant or adjuvant radiotherapy conferred to a better clinical outcome. Propensity score matching was used to control for confounding features.

**Results:**

Thirty thousand two hundred and forty-nine patients were included in this study (21,295 vs 8954 in surgery and adjuvant radiotherapy group); 1150 patients were matched in two groups. The median survivals in the surgery (S) group and adjuvant radiotherapy (S + R) group were 24 and 21 months, respectively. The 1-, 3-, and 5-year overall survival (OS) rates in the S group and S + R group were 68%, 40%, 31%, and 75%, 30%, 20%, respectively (*p* < 0.001), and the median OS was 22 and 25 months in S and S + R group after PSM, the former 1-, 2-, 3-, and 5-year OS were 73%, 45%, 30%, and 19%, and the later were 81%, 52%, 37%, and 24% (*p* = 0.0015), respectively; stratified analysis showed patients whose carcinoma located at pancreatic head with II stage infiltrating duct carcinoma (22 vs 25, *p* = 0.0276), T4 adenocarcinoma (28 vs 33, *p* = 0.0022), N1 stage adenocarcinoma (20 vs 23, *p* = 0.0203), and patients with infiltrating duct carcinoma received regional resection (23 vs 25, *p* = 0.028) and number of resected lymph node were ≥ 4 (22 vs 25, *p* = 0.009) had better OS after additional radiotherapy than surgery alone. Patients with pancreatic body/tail carcinoma III stage adenocarcinoma (13 vs, *p* = 0.0503) and T4 adenocarcinoma (14 vs, *p* = 0.0869) had survival advantage within 24 months for additional radiotherapy. However, patients with T2 stage adenocarcinoma located in pancreatic body/tail had better OS in surgery group than that in R + S group.

**Conclusions:**

Additional radiotherapy may contribute to improved prognosis for patients with pancreatic head II stage infiltrating duct carcinoma, III stage adenocarcinoma, T4 stage carcinoma, N1 stage adenocarcinoma, regional resection, or number of lymphadenectomy ≥ 4 in infiltrating duct carcinoma. A specific subgroup of patients with specific stage and histological type pancreatic cancer should be considered for additional radiotherapy.

**Supplementary Information:**

The online version contains supplementary material available at 10.1007/s12094-021-02671-0.

## Introduction

Pancreatic cancer is the seventh leading cause of cancer-related mortality, with an estimated 458,918 of new cases and 432,242 of deaths worldwide in 2018 [1]. It is predicted that pancreatic cancer will become the third cause of cancer-related death in the future [2]. Unfortunately, a multimodal therapy has not been able to improve pancreatic cancer patients’ prognosis. Surgery remains the most important treatment, but only 10–15% of patients are candidates for potentially curative resection, given that most patients would have atypical clinical symptoms and therefore present at the late advanced stage of the disease and not amenable for curative surgery. At present, the 5-year survival rate for pancreatic cancers is poor at less than 20%. Adjuvant radiotherapy and chemotherapy have been shown to improve the survival outcome of patients with pancreatic cancers, while neoadjuvant radiotherapy as well as intraoperative radiotherapy have also contributed to the improved prognosis. However, patient selection of suitability for additional radiotherapy or the optimal timing between the application of radiotherapy and surgery remains unclear.

To investigate the impact of adjuvant radiotherapy in patients with pancreatic cancer after surgical resection, we retrospectively analyzed patients from 18 registered institutions in the Surveillance Epidemiology and End Results (SEER) linked database. We aimed to determine the characteristics of patients that would benefit from adjuvant radiotherapy, and whether neoadjuvant or adjuvant radiotherapy conferred to a better clinical outcome.

## Patients and methods

### Data sources

The SEER*Stat software (version8.3.5) was used to retrieve data from the SEER database containing the SEER 18 registries (November 2018 submission). These registries covered approximately 90% of the US population (based on 201 censuses) from 1973 to 2016.

### Patients

SEER patients were diagnosed pancreatic malignancies with site code C25.0-c25.9, and with the International Classification of Disease for Oncology, Third Edition (ICD-O-3), histological classification codes of 8000 and 9260 were included in our study. Data on diagnosis confirmation, American Joint Committee on Cancer (AJCC) 7th Editor stage, T, N, M stage, histological grade, primary site surgery, regional lymph-node surgery, the sequence of surgery and radiotherapy, chemotherapy, overall survival in months, and survival status were collected. Meanwhile, patient’s basic demographics including age, pancreatic cancer diagnosis, and race were also gathered. Patients were excluded if data on survival or any survival status were not available.

Patients who underwent surgery were compared with patients who received surgery and radiotherapy. To identify the characteristics of patients that would benefit from adjuvant radiotherapy, the association of additional radiotherapy on survival with varying tumor histology, AJCC stage, age, race, primary site, grade, surgery primary site, regional lymph-node surgery, and chemotherapy were analyzed. Covariates of interest included patient-related factors (age, gender, and race), disease-level factors (primary site, stage, grade, and histologic classification), and treatment-related factors (surgery methods, radiotherapy, the sequence between surgery and radiotherapy, chemotherapy, and diagnosis confirmation).

### Statistics

The Fisher Exact Probability and ANOVA test were used to compare differences between categorical variables and continuous variables, respectively. The Cox proportional hazard regression model and Kaplan–Meier method were used to determine the effects of variables on OS and to visualize the survival curves, which were adjusted for other significant prognostic factors Propensity scores matched (PSM) were performed based on the 1:1 nearest-neighbor algorithm and logistic regression, with group (S, S + R) as the classified variable and other variables (age, gender, grade, primary site, ICD-0-3, AJCC stage, AJCC T/N/M stage, surgery T/N, and chemotherapy) as the matched variates. The cases with missing value of the paired variables were removed for good performance. All calculations were performed with SPSS version 23.0 for Windows software (IBM Corporation, Chicago, IL) and EmpowerStat software 2.0, GraphPad prism 7.0. Statistical significance was set at 0.05.

## Results

A total of 243,417 patients with pancreatic malignancy were identified from the SEER data for the years of 1975–2016. Of these, 7864 patients did not have survival data, and 43,722 patients whose survival time was 0 month thus were excluded from our analysis. A total of 115,371 patients did not undergo surgery, and in 46,211 patients, surgical data were not available, and thus, these were also eliminated. Finally, 30,249 patients were included in our study. The selection of patients was shown in Figure S1. Four thousand four hundred and eighty-four patients without missing information during 2007–2016 underwent PRM and filtered 1150 pairs of patients for next analysis after PRM.

### Baseline characteristics of patients

Thirty thousand two hundred and forty-nine patients were included in this study, 21,295 were in the surgery group (S group), and 8954 were in the combined surgery and radiotherapy group (S + R group). The demographics of the two groups were shown in Table S1. Patients in the S group were significantly older and more likely to be female than the S + R group. There were more tumors located at pancreatic body and tail in the S group, whereas a higher number of pancreatic head tumors were found in the S + R group. Most histological types of adenocarcinomas, carcinoma, and infiltrating duct carcinoma were identified in the S + R group, while there were more neuroendocrine neoplasms recorded in the S group. Patients with local-stage (AJCC 7th stage I, T0-2, N0, M0) disease underwent local excision (including lymph-node biopsy) in the S group and patients in the S + R group were mostly with regional-stage (AJCC 7th stage II, T 3–4, N1) and received regional surgery as well as chemotherapy. There were no significant differences in follow time and histologic grade between the two groups. More patients were still alive in the S group at the last follow-up. Age, AJCC 7th stage, and T/N stage were significant difference after PSM, and other features were identical, as shown in Table S1.

### Analysis of survival and risk factor

**Fig. 1:**
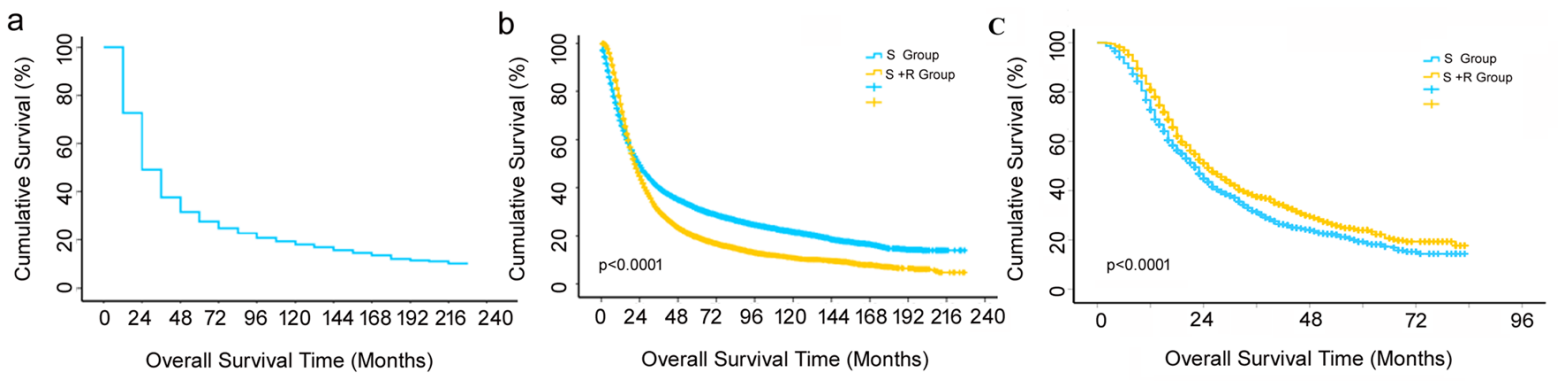
**a** Cumulative overall survival (OS) curves of all patients with pancreatic cancer; **b** OS curves of patients undergoing surgery alone (S) or surgery combined with radiotherapy (S + R). The log-rank test showed that the OS was significantly different between the two groups (*p* < 0.0001). S: surgery, S + R: surgery plus radiotherapy; **c** OS curves of patients undergoing surgery alone (S) or surgery combined with radiotherapy (S + R) after propensity score matching (PSM). The log-rank test showed that the OS was significantly different between the two groups (*p* < 0.0001). S: surgery, S + R: surgery plus radiotherapy

The median OS of all patients was 23 months, with the 1-, 3-, 5-year survival rate of 49%, 32%, and 25%, respectively. The median OSs in the S and S + R group were 24 and 21 months, respectively, as demonstrated in Fig. 1a. The 1-, 2-, 3-, and 5-year survival rates in the S group were 68%, 49%, 40%, and 31%, respectively, while the 1-, 2-, and 3-, 5-year survival rates in the S + R group were 75%, 45%, 30%, and 20% respectively; the differences in the two groups were statistically significance (*p* < 0.001), given that the 1-year OS appeared superior in the R + S group, whereas the 2-, 3-, and 5-year OSs were better in the S group, as demonstrated in Fig. 1b. However, the median OS was 22 and 25 months in S and S + R group after PSM, the former 1-, 2-, 3-, and 5-year OS were 73%, 45%, 30%, and 19%, and the later were 81%, 52%, 37%, and 24% (*p* = 0.0015), respectively; it was shown in Fig. 1c.

The univariate and multivariate analysis of the entire cohort showed that factors including age, gender, race, diagnosis year, primary site, ICD-0-3 classification, AJCC 7th stage, T, N, surgery primary tumor site (surgery T), surgery regional lymph node (surgery N), additional radiotherapy and chemotherapy were related to OS, as shown in Table S2. Factors such as patients in the R + S group (HR 0.88, 95% CI 0.85–0.91), additional chemotherapy (HR 0.82, 95% CI 0.79–0.85), diagnosis year between 1999 and 2006 (HR 0.88, 95% CI 0.80–0.98) and 2007–2016 (HR 0.78, 95% CI 0.69–0.87), pancreatic duct tumor (HR 0.99, 95% CI 0.96–1.03), neuroendocrine neoplasms (HR 0.17, 95% CI 0.16–0.19), mesenchymal tumor (HR 0.46, 95% CI 0.35–0.60), mucinous adenocarcinoma (HR 0.68, 95% CI 0.54–0.62), carcinoma (HR 0.48, 95% CI 0.43–0.53), and other histologic type (HR 0.56, 95% CI 0.52–0.60), and number of dissected lymph node with 1–3 (HR 0.91, 95% CI 0.84–0.98), and more than 4 (HR 0.81, 95% CI 0.76–0.87) were associated with lower risk of mortality. On the other hand, factors as independent predictors of higher mortality were age at 20–44 (HR 4.54, 95% CI 2.35–8.79), 45–64 (HR 6.31, 95% CI 3.27–12.17), 65–84 (HR 7.86, 95% CI 4.08–15.13), tumor overlapping lesion of pancreas (HR 1.1, 95% CI 1.02–1.17), adenosquamous carcinoma and squamous (HR 1.21, 95% CI 1.07–1.36), AJCC 7th stage II (HR 1.31, 1.15–1.5), III (1.73, 1.31–2.28), IV (2.53, 2.17–2.95) T2 (HR 1.49, 95% CI 1.31–1.71), T3 (HR 1.70, 95% CI 1.47–1.96), T4 (HR 1.67, 95% CI 1.28–2.18), N1 (HR 1.48, 95% CI 1.40–1.56), and resection expect for local removal. However, AJCC 7th T2 (HR 2.09, 95% CI 1.18–3.69), T3 (HR 2.58, 95% CI 1.45–4.56), T4 (HR 2.74, 95% CI 1.04–7.23), and N1 (HR 1.55, 95% CI 1.35–1.77) were correlated with adverse outcome, number of lymph-node dissection ≥ 4 (HR 0.70, 95% CI 0.52–0.94), and additional radiotherapy (HR 0.89, 95% CI 0.80–0.99) and chemotherapy (HR 0.54, 95% CI 0.40–0.72) were independent favorable prognostic factors experiencing PSM, as seen in Table S2. Histological grade was not associated with survive regardless of PSM.

### Stratification analysis by subgroup population

**Fig. 2:**
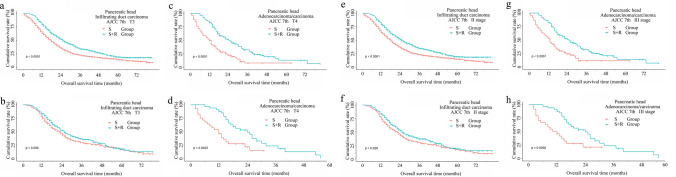
Cumulative overall survival (OS) curves of patients with pancreatic head cancer with sub-stratification analysis in surgery alone group and surgery plus radiotherapy group with a log-rank test, which demonstrated a sub-population of patients that benefited from additional radiotherapy. Top row (**a**, **c**, **e**, **g**), before matching. Bottom row (**b**, **d**, **f**, **h**), after matching

**Fig. 3:**
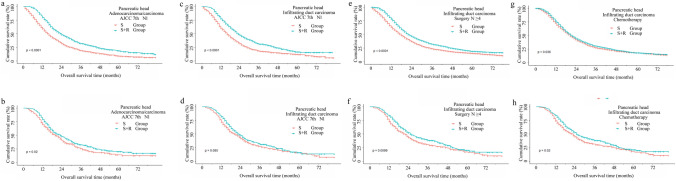
Cumulative overall survival (OS) curves of patients with pancreatic head cancer with sub-stratification analysis in surgery alone group and surgery plus radiotherapy group with a log-rank test, which demonstrated a sub-population of patients that benefited from additional radiotherapy. Top row (**a**, **c**, **e**, **g**), before matching. Bottom row (**b**, **d**, **f**, **h**), after matching

**Fig. 4:**
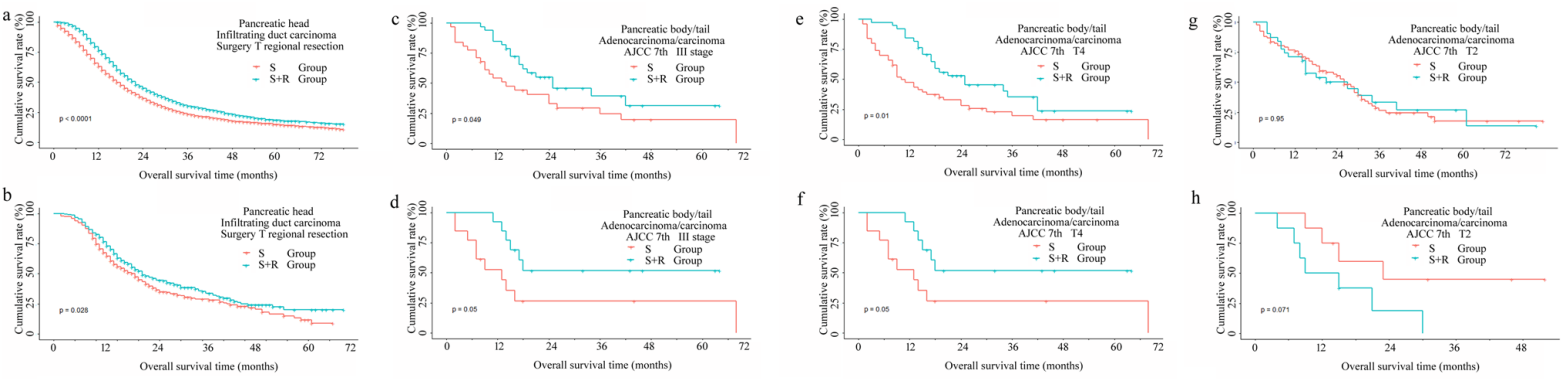
Cumulative overall survival (OS) curves of patients with pancreatic body/tail adenocarcinoma/carcinoma or infiltrating duct carcinoma, the sub-stratification analysis in surgery alone group, and surgery plus radiotherapy group with a log-rank test, which demonstrated a sub-population of patients that benefited from additional radiotherapy. Top row (**a**, **c**, **e**, **g**), before matching. Bottom row (**b**, **d**, **f**, **h**), after matching

To further investigate the optimum sub-population benefited from the additional radiotherapy, we carried out analysis in the data stratified by combination among primary site, histologic type, and other clinicopathologic features. In this sub-stratified evaluation, other tumor sites and histologic behavior were excluded due to small sample size, as well as definitive survival benefits from surgery alone and reverse risk for survival, as shown in Table S3 and Table S4. For patients with pancreatic head tumors, we found patients with stage T1 disease, T2 infiltrating duct carcinomas, lymphadenectomy < 4 with infiltrating duct carcinomas, without chemotherapy of adenosquamous/squamous showed no OS benefit from radiotherapy. Other patients with pancreatic head cancer had an improved survival following additional radiotherapy, as demonstrated in Table S3 and Figs. 2, 3. However, adjuvant radiotherapy improved the OS in subgroup included patients with AJCC 7th II stage infiltrating duct carcinoma (22 vs 25, *p* = 0.0276), T4 (12 vs 24, *p* = 0.0022), and N1(20 vs 23, *p* = 0.0203) adenocarcinoma/carcinoma (12 vs 24, *p* = 0.0022), infiltrating duct carcinoma received regional resection (23 vs 25, *p* = 0.028) (Fig. 4a, b), more than four lymph-node dissections (22 vs 25, *p* = 0.009), and chemotherapy (23 vs 25, *p* = 0.0202) after PSM (Fig. 3). Interestingly, we noticed three sub-population (AJCC 7th III stage adenocarcinoma, AJCC 7th T3 or N1 infiltrating duct carcinoma) had survival improvement when OS cutoff was 24 months (Fig. 3).

Meanwhile, stratified analysis was performed in patients with pancreatic body/tail before PSM. The beneficiaries form additional radiotherapy were less than those lesions located at pancreatic head, as displayed in Table S4. First, radiotherapy was helpful in OS for patients who had AJCC 7th I, II, III, T3, T4, N1 stage adenocarcinoma/carcinoma and infiltrating duct carcinoma. Second, surgical resection of primary tumor might improve OS, it demonstrated local resection followed radiotherapy made patients with adenocarcinoma/carcinoma and infiltrating duct carcinoma have better OS, and total pancreatectomy combined radiotherapy improved OS of cases with infiltrating duct carcinoma. On the other hand, the patient with adenocarcinoma/carcinoma and infiltrating duct carcinoma whose number of lymph nodes dissected ≥ 4 and patients with infiltrating duct carcinoma were removed 1–3 lymph nodes received radiotherapy had better prognosis. Finally, a survival advantage remained with chemotherapy for adenocarcinoma/carcinoma, as shown in Table S4. After PSM, patients with AJCC 7th III, T4 adenocarcinoma/carcinoma had survival advantage within 24 months from additional radiotherapy, as shown in Table S4 and Fig. 4. However, patients with T2 adenocarcinoma located in pancreatic body/tail had better OS in surgery alone than that in R + S group, as displayed in Table S4 and Fig. 4.

### The sequence of surgery and radiotherapy and survival, radio source

**Fig. 5:**
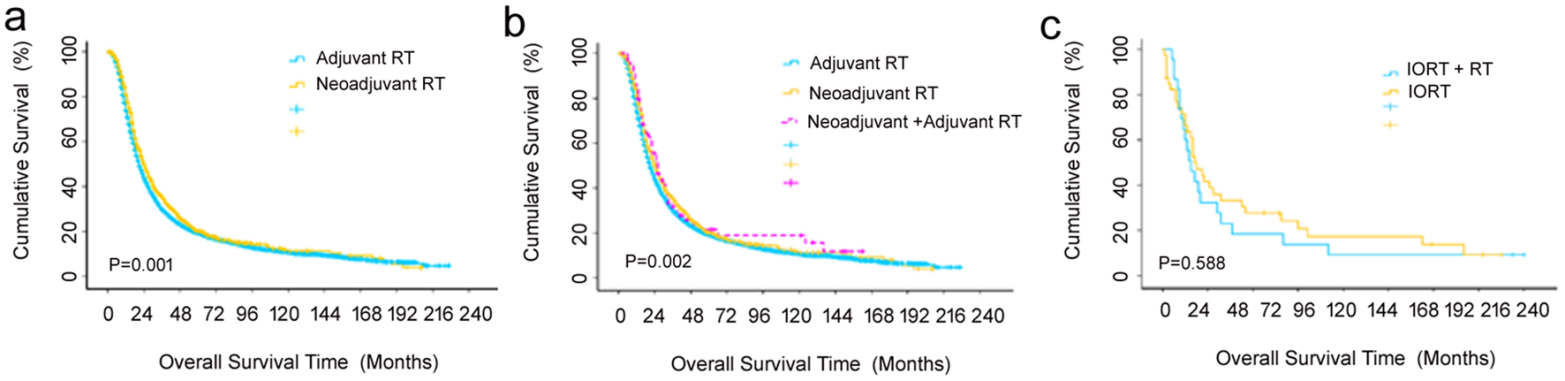
Cumulative overall survival (OS) curves of patients with pancreatic cancer, and the sub-stratification analysis of sequence between radiotherapy and surgery with a log-rank test. **a** Adjuvant and neoadjuvant radiotherapy (*p* = 0.001); **b** adjuvant, neoadjuvant, and both; **c** intraoperative radiotherapy plus radiotherapy and intraoperative therapy alone (*p* = 0.588)

Following the identification of a specific patient population that benefited from the additional radiotherapy in addition to surgery, the next concern involving the sequence between surgery and radiotherapy was addressed, as demonstrated in Table S5. A total of 7664 and 1099 patients received adjuvant and neoadjuvant radiotherapy, respectively. The median survival was 21 months (95% CI 20–22) vs 25 months (95% CI 23–27), while the 1-, 3-, and 5-year OS were 74%, 29%, and 20% vs 81%, 35%, and 20% in the adjuvant and neoadjuvant populations, respectively, for which the differences were statistically significant (*p* = 0.001), as shown in Fig. 5a. There were 76 patients that received radiotherapy both before and after surgery, with the median survival of 26 months (95% CI 20–32) and the 1-, 3-, and 5-year OS of 85%, 32%, and 19%, respectively, which were significantly better than those having either radiotherapy before or after surgery only (*p* = 0.002), as shown in Fig. 5b. Finally, a small proportion of patients had intraoperative radiotherapy with or without further adjuvant radiotherapy, for which the survival difference between these two subgroups was not statistically significant (*p* = 0.588), as shown in Fig. 5c.

## Discussion

Adjuvant chemotherapy or radiotherapy in combination with surgery has been the standard of treatment for pancreatic cancer for decades [3]. External beam radiotherapy is one of the most important treatment as it contributes to better survival [3–16]. In this study, we determined the potential patient population who might be benefited from having additional radiotherapy. Our primary survival analysis indicated that the benefit from additional radiotherapy was critically time-dependent, and if extending the time window of more than 24 months, the treatment benefit from extension could be reverse. However, the multivariate analysis showed that additional radiotherapy was an independent prognostic factor for OS (HR 0.89, 95% CI 0.86–0.92, *p* < 0.0001). Moreover, earlier studies have demonstrated that patients treated with radiotherapy had significantly better survival than those without [7, 8, 11–21]. The conflicting results we observed might be attributed to the differences in the baseline characteristics of patients as a result of retrospective selection bias. Patients who had received additional radiotherapy were of a poor prognostic group with several high-risk factors, including advanced stage, anatomic site, and unfavorable histologic classification [22–29]. Despite these, our study showed that patients with the above unfavorable features could still benefit from additional radiotherapy.

Pancreatic head has been shown to be the most common anatomical site for cancer followed by the pancreatic body and tail, with the latter displaying poorer survival than pancreatic head lesions [29]. Adenosquamous carcinoma of the pancreas has a higher prevalence in the pancreatic tail and associated with poor survival [22]. In our study, the sample size of adenosquamous/squamous carcinoma was small and the patients with pancreatic body/tail cancer did not appear to benefit from additional radiotherapy. Patients with pancreatic neuroendocrine cancer have a better life expectancy [24, 25], and resection of the primary tumor is associated with longer survival in stages I–III as well as stage IV tumors [26]. Therefore, representing as the third most common tumor type, patients with pancreatic neuroendocrine cancer in our study had extremely good survival prognosis after surgical resection. A similarly favorable survival outcome was observed in patients with mesenchymal tumors or mucinous adenocarcinoma.

To date, pancreatic adenocarcinoma/carcinoma and infiltrating duct carcinoma are still the most difficult to treat. In addition, small pancreatic cancers have been associated with poorer survival prognosis and an extremely high rate of nodal involvement, for which they should not be regarded as early-stage cancers [30]. Similar findings were observed in our study, in which additional radiotherapy significantly contributed to improving survival in patients with stage II infiltrating duct carcinoma located in pancreatic body/tail. However, based on the AJCC 7th classification, stage I/II disease indicates a T1/T2 tumor that is localized within the pancreas. Our data showed that additional radiotherapy for patients with T2 adenosarcoma in the pancreatic body/tail had had adverse survival benefit within 2 years. In the current National Comprehensive Cancer Network (NCCN) guideline (https://www.nccn.org), an early-stage pancreatic carcinoma refers to a resectable tumor. Neoadjuvant therapy can be considered in a clinical trial or with chemotherapy alone, or induction chemotherapy followed by chemotherapy, especially in the presence of high-risk factors. In addition, chemoradiation should not be given to patients after surgery except in clinical trials [31]. Importantly, most clinical trials have been focusing on comparing chemotherapy with or without chemoradiotherapy, and a few trials have examined the effectiveness of radiotherapy alone. Our study demonstrated that patients with stage II or T2 disease had an improved survival from additional radiotherapy, suggesting that those sub-population patients especially with high-risk features and did not receive neoadjuvant treatment could be considered for adjuvant radiotherapy. The AJCC 8th stage system was prior to current stage for involved in clear T stage, [32] and future prospective clinical study should be designed to take into account patients with diseases of early stages.

Our study demonstrated that locally advanced (T3/4, N^+^) pancreatic adenocarcinoma/carcinoma or infiltrating duct carcinoma had improved survival prognosis following additional radiotherapy, with particular tumor location. Several studies have found adjuvant treatment after surgery contributed to improved survival in advanced pancreatic cancer [3–7, 11, 13, 33, 34]. Other clinical trials have confirmed neoadjuvant treatment was prior to adjuvant treatment [3, 5, 8, 14, 21, 35]; in fact, most patients preferentially received resection; more than 80% of patients were in the station in current study (Table S5). Surprisingly, our study showed that patients who benefited from radiotherapy were those who received chemotherapy, and this was in keeping with the results of previous studies which demonstrated that chemoradiotherapy or chemotherapy sequencing with radiotherapy was beneficial for survival improvement. An increased number of lymph-node dissection have been shown to be a poor prognostic factor for OS [36], with higher lymph-node retrieval associated with a higher proportion of positive invasion. Our study showed that patients with more than four lymph-node dissections in patients with infiltrating duct carcinoma had a favorable survival when treated with additional radiotherapy. Therefore, radiotherapy can improve cancer survival compared with cancer-directed surgery without radiation in patients with infiltrating duct adenocarcinoma of the pancreas. Patients with advanced disease regardless of tumor location should be considered for radiotherapy and chemotherapy.

## Conclusions

Patients with pancreatic cancer of specific histological types (adenocarcinoma/carcinoma, infiltrating duct carcinoma, adenosquamous carcinoma, and squamous), anatomical location, and advanced stage have poor survival, and therefore, additional radiotherapy may contribute to an improved survival prognosis. A specific subgroup of patients with AJCC 7th stage II or T3 infiltrating duct carcinoma and N1 adenocarcinoma located pancreatic head had survival benefit from additional radiotherapy. Patients with adenocarcinoma/carcinoma stages AJCC 7th III, T4 whose lesions located pancreatic body/tail should be considered for additional radiotherapy. Our findings highlight the positive role of radiotherapy in highly selected patients with pancreatic malignancy, suggesting the need for future prospective randomized control study to confirm these findings before recommending such treatment to patients.

## Supplementary Information

Below is the link to the electronic supplementary material.

Supplementary file1 (TIF 265 KB)

Supplementary file2 (XLSX 38 KB)

## Data Availability

Original data are available at Surveillance Epidemiology and End Results (SEER) database. (https://seer.cancer.gov/data-software/documentation/seerstat/).

## Abbreviations

HCC: Hepatocellular carcinoma
OS: Overall survival
SEER: Surveillance epidemiology and end results
MS: Median survival
S: Surgery
R: Radiotherapy
ICD-3: International classification of disease for oncology, third edition
AJCC: American Joint Committee on Cancer
HR: Hazard rate
CI: Confidence interval
