# Supplementary figures and images for: The role of radiotherapy for pancreatic malignancies: a population-based analysis of the SEER database
Source: Clin Transl Oncol. 2021 Jul 4;24(1):76–83. doi: 10.1007/s12094-021-02671-0 (PMC8732853; doi:10.1007/s12094-021-02671-0)

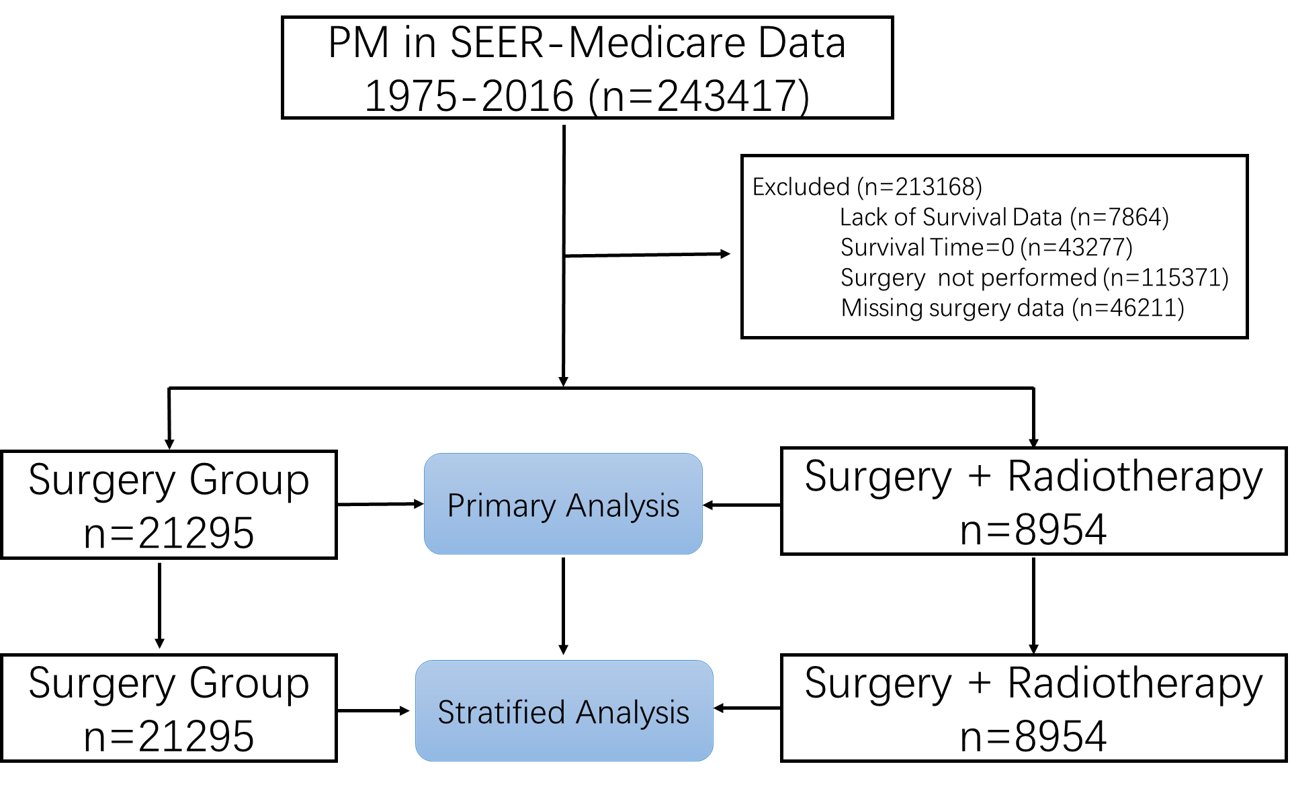

Supplement: Supplementary file 1 — Supplementary file1 (TIF 265 KB) [file 12094_2021_2671_MOESM1_ESM.tif]
